# Transoral robotic surgery with radial forearm free flap reconstruction: case control analysis

**DOI:** 10.1186/s40463-017-0196-0

**Published:** 2017-03-14

**Authors:** Vincent L. Biron, Daniel A. O’Connell, Brittany Barber, Jessica M. Clark, Colin Andrews, Caroline C. Jeffery, David W. J. Côté, Jeffrey Harris, Hadi Seikaly

**Affiliations:** 1 Department of Surgery, Division of Otolaryngology-Head & Neck Surgery, University of Alberta, 8440-112 st, 1E4 Walter Mackenzie Centre, Edmonton, AB T6G 2B7 Canada; 2 Alberta Head & Neck Centre for Oncology and Reconstruction, Edmonton, AB Canada; 3 Faculty of Medicine and Dentistry, Undergraduate Medical Education, Edmonton, AB Canada

**Keywords:** Oropharyngeal cancer, Transoral robotic surgery, Mandibulotomy, Radial forearm free flap, Human papillomavirus

## Abstract

**Background:**

The resection of large oropharyngeal tumors traditionally involves a lip-splitting mandibulotomy for adequate margin visualization and free flap reconstruction of the surgical defect. Transoral robotic surgery (TORS) has emerged as a technique that can resect large and complex oropharyngeal tumors, avoiding a lip-splitting approach. The aim of this study is to compare the lip-splitting mandibulotomy approach versus TORS for the management of advanced stage oropharyngeal carcinomas.

**Methods:**

Prospectively collected data from 18 patients with advanced stage oropharyngeal squamous cell carcinoma (OPSCC) who received TORS with radial forearm free flap reconstruction (RFFF) was compared to a matched cohort of 39 patients who received a lip-splitting mandibulotomy and RFFF. Patients were matched for stage, p16 positivity, smoking, age and gender. Length of hospital stay (LOHS), tracheostomy decanulation time, operative time, surgical margin status, and post-operative complications were compared between groups.

**Results:**

Patients who received TORS with RFFF had a significantly lower mean LOHS, compared to patients who were treated by lip-splitting mandibulotomy and RFFF (14.4 vs 19.7 days, *p* = 0.03). No significant differences were seen between groups in terms of operative time, tracheostomy decannulation time, margin positivity and post-operative complications.

**Conclusion:**

TORS with radial forearm free flap reconstruction is a safe, effective and cost-saving alternative to the lip-splitting mandibulotomy approach for the treatment of advanced stage OPSCC.

## Background

Oropharyngeal squamous cell carcinoma (OPSCC) is caused by tobacco smoking, alcohol and oncogenic human papillomavirus (HPV). Over the past decade, the incidence of HPV-related OPSCC has been rising worldwide [1–7]. When compared to non-HPV related OPSCC, HPV-OPSCC is molecularly distinct and patients with these tumors have improved survival outcomes regardless of treatment modality [8–20].

The treatment of advanced stage OPSCC has been a subject of controversy in recent years, as comparable survival outcomes can be achieved with either chemoradiation or primary surgery for HPV-positive tumors. The increasing use of transoral robotic surgery (TORS) since its FDA approval in 2009 has paralleled a paradigm shift towards primary surgical treatment of OPSCC [9, 21–25]. Several reports have shown favorable oncologic outcomes with TORS, while minimizing morbidity and disfigurement associated with mandibulotomy approaches [26–28]. In comparison to chemoradiation, treatment of OPSCC patients with TORS has also been shown to result in better functional outcomes and decreased cost [21, 22, 29–31].

TORS has proven efficacy in the resection of small primary (T1 and T2) oropharyngeal tumors, however, with increasing use, the applications of this tool has expanded. Recent reports from several independent centers have described the use of TORS for the resection of more advanced oropharyngeal cancers, those requiring free flap reconstruction [32–40], in cases traditionally approached by lip-splitting mandibulotomy. TORS with free flap reconstruction (TORS-FF) is thought to be a safe and less invasive alternative to the mandibulotomy approach. However, the literature describing TORS-FF is limited to case reports and small case series with no comparative group.

The goal of this study was to compare TORS to the lip-splitting mandibulotomy approach in the primary surgical treatment of advanced stage OPSCC in patients with planned free flap reconstruction.

## Methods

### Patient data

All OPSCC patients who received TORS and radial forearm free flap reconstruction (RFFF) from May 2015-July 2016 or lip-splitting mandibulotomy with RFFF from January 2006-July 2016 at the University of Alberta were included in the study. Patient demographics were obtained from prospectively collected databases through the Alberta Cancer Registry, with details verified in paper charts and electronic medical records. Smoking status was defined as having a greater than 10 pack year tobacco smoking history [11]. P16 status was obtained though standard pathology for TORS patients and from tissues microarray data previously reported for mandibulotomy patients [9]. Operative time was obtained from standardized nursing and anesthesia records, calculated from induction of general anesthesia to surgical case completion. Surgical margin status was obtained from final post-operative pathology. Length of hospital stay was calculated from the start of surgical procedure to date of discharge based on standardized discharge criteria according to head and neck care protocols [41].

### Transoral robotic surgery

All patients received a prophylactic tracheostomy for airway safety [32, 33, 35, 38, 42] as well as a nasogastric tube placement. TORS was performed using the da Vinci S Surgical System (Intuitive Surgical, Sunnyvale, CA) at the beginning of the procedure with the exception of three patients who had ipsilateral N3 nodal disease. In N3 patients, the neck dissection was completed for safe exposure of the carotid artery, followed by TORS of the primary tumor. Oropharyngeal exposure was obtained using Crowe-Davis or Feyh-Kastenbauer mouth retractor and visualized with a 0- or 30- degree endoscope. TORS resection was performed using monopolar cautery and a Maryland dissector. Intraoperative frozen sections were taken from the ablative defect.

### Free flap reconstruction of TORS defect

Once the size of the oropharyngeal defect was known an appropriate sized free flap was elevated in the standard fashion. The radial forearm free flap was used for its versatility in reconstructing of multiple defects of the oropharynx including fasciocutaneous re-lining, soft palate repair as well as beaver-tail modifications for functional base of tongue reconstruction [43–46]. An analysis of the tumor extirpation defect was done based on subsite(s) resected, including percent resected of the soft palate, lateral pharyngeal wall/tonsil and base of tongue. If > 50% of the soft palate was resected functional soft palate reconstruction was initiated [45], and if > 50% of the base of tongue is resected a beavertail modified free flap was utilized [44]. Two patients had >50% of total tongue base resected and 1 patient had > 50% of soft palate resected. These oropharyngeal reconstructive techniques are comprehensively described elsewhere [46].

Trans-cervical and pharyngeal accesses were used for inferior repair of the pharynx and inset of the free flap. The ipsilateral suprahyoid muscles and nerves were preserved whenever possible but the ipsilateral hypoglossal and lingual nerves can be transected to provide wider access. The lingual nerve was transected and re-anastomosed following inset in 3 patients. These nerves should be reconstructed primarily or cable grafted, to limited the impact on post-operative speech and swallowing function [47].

The inferior portion of the flap was inset initially transcervically, though the lateral pharyngotomy The remainder of the flap was then inset via the oral cavity through a transoral approach. The robot was not used to inset any portion of the flap. Microvascular anastomosis to recipient vessels in the neck were performed after the inset of the flap was complete.

### Cost comparison

Surgical instrument costs were determined from nursing case logs for instrument use standard to TORS vs mandibulotomy approaches for oropharyngeal cancer resections. Physician billing costs were estimated from standard surgeon and anesthesia billings according to the Alberta Health Services Schedule of Medical Benefits (http://www.health.alberta.ca/professionals/SOMB.html). Hospital stay cost estimates were obtained from Alberta Health Services cost estimates (http://obrieniph.ucalgary.ca/health-econ/research-reports).

### Data analysis

Patients who received a lip-splitting mandibulotomy and RFFF were systematically matched to patients who received TORS with RFFF. Patients were fist matched by exact T stage and p16 status, followed by closest matching possible for smoking status, age, gender and nodal stage. Statistical comparisons of group variables were performed in SPSS version 22 (Chicago, IL, USA) using Chi-Square or Mann Whitney U tests where appropriate.

## Results

### Patient characteristics

**Fig. 1:**
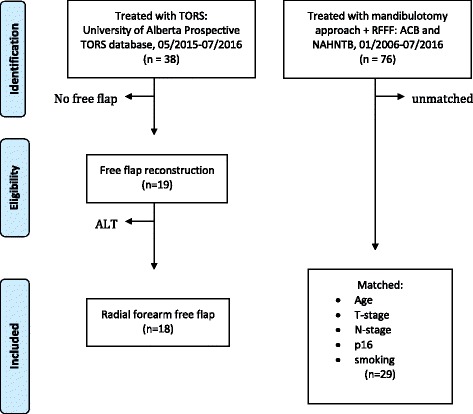
Summary of oropharyngeal cancer patients selected for inclusion in this study

From May 2015 to July 2016, 38 patients received TORS at the University of Alberta, of which 18 received a radial forearm free flap (RFFF) reconstruction and were included in this study. These patients were compared to a historical cohort of 76 OPSCC patients who were treated with primary surgery using a lip-splitting mandibulotomy approach and RFFF (Fig. 1). Patients met criteria for free flap reconstruction of TORS defects if they had one or more of the following adverse features as previously described [37]. 1) >50% palate defect, 2) pharyngo-cervical communication and/or 3) exposed pharyngeal internal carotid artery. All patients in the TORS or mandibulotomy cohorts met the above criteria (all class III/IV) and therefore met indications for free flap reconstruction.

Twenty-nine patients who received a mandibulotomy were identically matched to the TORS patients for p16 positivity, smoking and T-stage, and closely matched for age (+/-10 years), sex, tumor subsite and nodal status (Fig. 1 and Table 1). In comparing matched patients who received TORS + RFFF vs mandibulotomy + RFFF, there were no statistically significant differences between these groups with regard to age, sex, p16 positivity, primary tumor subsite, T-stage and N-stage (Table 1).

**Table 1:** Matched demographic, exposure and tumor characteristics of patients with oropharyngeal squamous cell carcinoma in this study

Variable	TORS (*n* = 18)	Mandibulotomy (*n* = 29)	*P*
Age, years	59.6	57.6	0.69
Sex, % M	66.7	80.7	0.31
Smoking status (%)	10 (55.5)	17 (58.6)	0.67
P16 positivity (%)	16 (88.9)	25 (93.1)	0.61
Tumor subsite (%)			
*Tonsil*	13 (72.2)	16 (55.2)	0.36
*Base of Tongue*	5 (27.8)	13 (44.8)	
Pathologic stage (%)			
*T1*	5 (27.8)	7 (26.9)	0.96
*T2*	10 (55.5)	17 (58.6)	
*T3*	3 (16.7)	5 (19.2)	
*N0*	1 (5.6)	1 (3.8)	
*N1*	1 (5.6)	3 (11.5)	0.68
*N2*	13 (72.2)	23 (79.3.)	
*N3*	3 (16.7)	2 (7.7)	

### Operative outcomes

No TORS cases were converted to open, lip-splitting mandibulotomy. Negative intra-operative frozen sections were obtained for all patients treated with TORS-FF. Two (6.9%) patients treated with a lip-splitting approach had positive margins reported on post-operative pathology. The operative time was similar for patients treated with a TORS (15.0 h) vs mandibulotomy (15.5 h) approach (Table 2).

**Table 2:** Outcomes of oropharyngeal cancer patients treated with TORS vs mandibulotomy and radial forearm free flap reconstruction

Outcome	TORS	Mandibulotomy	*P*
Operative time	15.0	15.5	0.77
LOHS (days)	14.4	19.7	0.03
Positive margins (%)	0	6.9	0.52
ICU stay (days)	1.9	2.0	0.76
Decannulation (days)	7.5	9.1	0.97
G-tube 1 month (%)^a^	16.6	13.7	0.68
G-tube 12 months (%)^b^	5.5	13.7	0.50

*ICU* intensive care unit; *LOHS* length of hospital stay

^a^G-tube dependency and readmissions to hospital reported for up to 30 days post-discharge

^b^1 year g-tube rates not available for all given the study end date (July 2016) but is available to all patients who did receive a g-tube

### Post-operative outcomes

Patients treated with TORS had a significantly shorter length of hospital stay compared to mandibulotomy patients (14.4 vs 19.7 days, *p* = 0.03). No significant differences were seen between these groups in terms of post-operative intensive care unit stay, time to decannulation or gastrostomy tube dependency (Table 2).

### Adverse events

Although valid statistical comparisons cannot be made between groups, patients treated with TORS experienced fewer complications overall (Table 3). Patients had comparable numbers of post-operative hematoma, abscess, chyle leak and blood loss requiring post-operative packed red blood cell transfusion. In the mandibulotomy group however, three patients experienced airway obstruction post-tracheostomy decannulation (requiring re-cannulation), which did not occur in TORS patients. There were no free flap failures in either group. No intraoperative or perioperative fatalities occurred.

**Table 3:** Adverse events in patients receiving TORS vs mandibulotomy and radial forearm free flap reconstruction

Event	TORS	Mandibulotomy
Hematoma	1	1
Abscess	2	3
Chyle leak	1	1
Blood transfusion^a^	3	5
Airway obstruction^b^	0	3
Pulmonary embolism	0	1
Stroke	0	1
Fistula	0	0
*TOTAL*	*7*	*15*

No significant differences were seen between groups

^a^Blood transfusions were measured as either intra-operative or post-operatively up to the point of discharge from hospital

^b^Airway obstruction post-tracheostomy decannulation requiring further intervention

### Cost comparison

Comparison of cost estimates for TORS vs mandibulotomy approaches showed reduced cost of surgical instruments, physician billings and hospital stay associated with TORS (Table 4). Overall, the TORS approach is estimated to result in a cost reduction of $ 6409.98 per case.

**Table 4:** Cost comparison of TORS vs mandibulotomy and radial forearm free flap reconstruction

Items	TORS	Mandibulotomy
Surgical instruments^a^		
Robotic arms (x2)	$ 1109.72	-
Robotic drapes	$ 469.67	-
Plates and screws	-	$ 1072.58
Saw blades and tubing	-	$ 693.53
Physician billings^b^	$ 181.19	$ 235.25
Surgical ward stay (mean)	$ 16,761.6	$ 22,930.8
*Totals*	*$ 18, 522.18*	*$ 24,932.16*

Cost shown per case in Canadian dollars. Operating time and intensive care unit stays were not statistically different between both groups and is not shown in the analysis

^a^Only surgical items that are different between both cases are included. Cost of non-disposable items such as the Da Vinci robotic system (purchased prior to the study for non-head and neck robotic surgery) and drills/saws are not included

^b^Includes only billings that are different between both cases, for anesthesia and surgeon codes as per the 2014–2016 Alberta Health Services Schedule of Medical Benefits

## Discussion

TORS has been mainly used for the resection of small (T1 or T2) OPSCCs with the resulting defect left to heal secondarily or by primary closure. Recently, a number of reports have described the use of TORS for the resection of larger tumors, traditionally approached by lip-splitting mandibulotomy followed with free flap reconstruction [32–40]. To date, this study reports outcomes on the largest cohort of OPSCC patients treated with TORS and free flap reconstruction and provides the best available evidence for this approach.

TORS with free flap reconstruction is a recent surgical advancement with literature describing this procedure limited to case reports and small case series ranging from one to eleven patients [32–40, 42]. The most common post-TORS free flap reported in the literature is the radial forearm (*N* = 37), followed by anterolateral thigh (*N* = 5) and vastus lateralis (*N* = 1). The radial forearm free flap is generally the first option for oropharyngeal reconstruction [44, 45, 48–52] unless it is contraindicated. Given the pliability of this flap, it is an excellent option for reconstruction of TORS oropharyngeal defects, whereby insetting is challenging with more limited access.

The overall surgical time of this approach was comparable to matched mandibulotomy cases (15.0 vs 15.5 h, *p* = 0.77) even though TORS resection of the primary tumor may result in a more challenging free flap inset. TORS-FF operative time reported in this study was also similar to data in the literature, ranging from 12.25 to 17.5 h [38, 40]. In our experience, additional time is required for TORS set-up and free flap insetting is counter balanced by avoiding a mandibulotomy and plate reconstruction.

Traditionally a lip-splitting mandibulotomy was required for oncologic resection of oropharyngeal tumors followed by free tissue reconstruction in most cases. This time proven approach provides safe and effective surgical access, but is associated with delayed recovery of oral function and obvious facial scarring [34, 53]. TORS offers the advantage of superb visualization as demonstrated by the reduced positive margin rate and precise instrumentation to perform large oropharyngeal resections in a less invasive and cosmetically superior fashion. In our case-matched cohort, patients who received TORS-FF were discharged from hospital an average of 5.3 days earlier than patients who were treated with mandibulotomy. Reasons for decreased LOHS in TORS patients could not be unequivocally ascertained but these data are consistent with the literature [33]. We hypothesize that TORS-FF patients meet discharge criteria earlier due to a number of factors such as edema, pain control and swallowing but this requires additional investigation. This reduction in LOHS may also result in significant health care cost savings.

Recent studies suggest TORS may provide superior swallowing outcomes for patients with OPSCC. A review of the US Nationwide Inpatient Sample reported a significant decrease in g-tube rates (0% vs 19%) in OPSCC patients treated with TORS vs other surgical approaches [30]. Sharma et al. reported lower g-tube rates in OPSCC patients treated with TORS vs a T-stage matched cohort treated with chemoradiotherapy [31]. In case series of patients receiving TORS-FF for OPSCC, one group reported 44% (4/9) of patients to be g-tube dependent at 1 year [32], while another group reported only 9% (1/11) patients temporarily required a g-tube [37]. Our TORF-FF patients had comparable rates of g-tube dependency (16.6%) to patients who received a mandibulotomy. We are performing further follow-up assessments of function in a larger group of patients receiving TORS-FF to evaluate the effects of surgery on swallowing.

A number of studies have demonstrated lower positive margin resection rates with TORS vs other surgical approaches, which potentially translates into improved local disease control [21, 22, 26, 27] and de-intensification of adjunctive treatment. Two large multi-institutional studies (*N* = 177 and *N* = 410) estimated positive margins rates from TORS to be 4.3 to 9.9% with close margins (1–5 mm) in 21.0% of patients receiving TORS [26, 27]. Our study demonstrated low rates of positive margins in TORS-FF (0%) and mandibulotomy patients (6.9%). Our TORS approach aims to take ≥1 cm margins, which can be performed safely in the setting of planned free flap reconstruction. Although long-term follow-up would be required to verify the oncologic outcomes associated with the TORS-FF, this data suggests at least equivalent local disease control can be achieved with this approach.

A number of limitations should be considered when interpreting results from this study. Bias between comparative groups was minimized using a case-control study design, however, data from the lip-splitting mandibulotomy cohort was obtained retrospectively. Cost comparisons are based on Alberta Health Services mean costing data and only includes estimated costs associated with the surgical procedues and hospital stay. Additional costs such as patient/caregiver travel, parking, childcare and loss of employment could not be accurately calculated in this study and are therefore not included. Some confounders may have influenced the LOHS between groups, given the implementation of head and neck post-operative care pathways in 2015. As the majority of mandibulotomy patients being compared received post-operative care prior to 2015, the LOHS in this group could be lower. In the mandibulotomy patients who were treated within the formally implemented care pathway the mean LOHS was 16 days, still higher than TORS patients. In addition, this study was performed in a single centre with a high-volume experience in head and neck oncologic and surgery and reconstruction. Further prospective and multi-centre studies are suggested to validate our findings.

## Conclusion

TORS with radial forearm free flap reconstruction is a safe, effective and potentially cost-saving alternative to the lip-splitting mandibulotomy approach for the treatment of advanced stage OPSCC.

## Abbreviations

ALT: Anterolateral thigh
FF: Free flap
HPV: Human papillomavirus
ICU: Intensive care unit
LOHS: Length of hospital stay
OPSCC: Oropharyngeal squamous cell carcinoma
RFFF: Radial forearm free flap
TORS: Transoral robotic surgery
